# Supplementary material for: The Impact of Myosteatosis Percentage on Short-Term Mortality in Patients with Septic Shock
Source: J Clin Med. 2022 May 27;11(11):3031. doi: 10.3390/jcm11113031 (PMC9181588; doi:10.3390/jcm11113031)
Supplement: Supplementary file 1 [file jcm-11-03031-s001.zip › jcm-1692743-supplementary.pdf]

Table S1. Baseline characteristics of the male patients

Characteristics	Total (n = 527)	Survivor (n = 433)	Non-survivor (n = 94)	*p*-value
Age	67.0 (59.0 – 73.0)	67.0 (59.0 – 74.0)	65.0 (58.0 – 71.0)	0.12
Past illness				
HTN	175 (33.2)	144 (33.3)	31 (33.0)	0.96
DM	153 (28.8)	130 (30.0)	22 (23.4)	0.20
CAD	60 (11.4)	54 (12.5)	6 (6.4)	0.09
Pulmonary disease	41 (7.18)	30 (6.9)	11 (11.7)	0.12
Malignancy	222 (42.1)	177 (40.9)	45 (47.9)	0.21
Hematologic disorder	38 (7.2)	27 (6.2)	11 (11.7)	0.06
CKD	31 (5.9)	29 (6.7)	2 (2.1)	0.09
LC	100 (19.0)	76 (17.6)	24 (25.5)	0.07
Site of infection				
Unknown	49 (9.3)	39 (9.0)	10 (10.6)	0.62
Lung	100 (19.0)	77 (17.8)	23 (24.5)	0.13
Urinary tract	46 (8.7)	42 (9.7)	4 (4.3)	0.09
Intra-abdomen	82 (15.6)	59 (13.6)	23 (24.5)	< 0.01
Hepato-biliary-pancreas	237 (45.0)	204 (47.1)	33 (35.1)	0.03
Blood stream	21 (4.0)	13 (3.0)	8 (8.5)	0.01
Lactate level	4.0 (2.0 – 6.0)	3.6 (1.9 – 5.5)	5.4 (2.7 – 9.2)	< 0.01
SOFA score	7.0 (5.0 – 10.0)	7.0 (5.0 – 10.0)	9.0 (6.0 – 12.0)	< 0.01
APACHE score	16.0 (11.0 – 20.0)	15.0 (11.0 – 19.0)	18.0 (14.0 – 22.8)	< 0.01

Sarcopenia	328 (62.2)	268 (61.9)	60 (63.8)	0.73
Myosteatosis	419 (79.5)	339 (78.3)	80 (85.1)	0.14

Data are presented as n (%) or median (interquartile range).

Abbreviations: HTN, hypertension; DM, diabetes mellitus; CAD, coronary artery disease; CKD, chronic kidney disease; LC, liver cirrhosis; SOFA, sequential organ failure assessment; APACHE, acute physiology and chronic health evaluation.

Table S2. Baseline characteristics of the female patients

Characteristics	Total (n = 369)	Survivor (n = 317)	Non-survivor (n = 52)	*p*-value
Age	67.0 (57.0 – 76.0)	68.0 (57.8 – 77.0)	65.5 (56.8 – 77.3)	0.62
Past illness				
HTN	124 (33.6)	109 (34.4)	15 (28.8)	0.43
DM	78 (21.1)	65 (20.5)	13 (25.0)	0.46
CAD	17 (4.6)	15 (4.7)	2 (3.8)	0.78
Pulmonary disease	6 (1.6)	4 (1.3)	2 (3.8)	0.17
Malignancy	155 (42.0)	126 (39.7)	29 (55.8)	0.03
Hematologic disorder	20 (5.4)	14 (4.4)	6 (11.5)	0.04
CKD	20 (5.4)	16 (5.0)	4 (7.7)	0.44
LC	39 (10.6)	32 (10.1)	7 (13.5)	0.46
Site of infection				
Unknown	33 (8.9)	28 (8.8)	5 (9.6)	0.86
Lung	47 (12.7)	35 (11.0)	12 (23.1)	0.02
Urinary tract	95 (25.7)	85 (26.8)	10 (19.2)	0.25
Intra-abdomen	59 (16.0)	49 (15.5)	10 (19.2)	0.49
Hepato-biliary-pancreas	121 (32.8)	107 (33.8)	14 (26.9)	0.33
Blood stream	18 (4.9)	13 (4.1)	5 (9.6)	0.09
Lactate level	3.0 (1.5 – 5.7)	2.8 (1.4 – 5.1)	5.9 (2.6 – 8.7)	< 0.01
SOFA score	7.0 (5.0 – 9.0)	6.0 (5.0 – 8.0)	11.0 (6.0 – 14.0)	< 0.01
APACHE score	15.0 (11.0 – 20.0)	14.0 (11.0 – 19.0)	20.5 (15.0 – 30.3)	< 0.01

Sarcopenia	239 (64.8)	206 (65.0)	33 (63.5)	0.83
Myosteatosis	313 (84.8)	268 (84.5)	45 (86.5)	0.71

Data are presented as n (%) or median (interquartile range).

Abbreviations: HTN, hypertension; DM, diabetes mellitus; CAD, coronary artery disease; CKD, chronic kidney disease; LC, liver cirrhosis; SOFA, sequential organ failure assessment; APACHE, acute physiology and chronic health evaluation.
